# Opposing effects of BRCA1 mRNA expression on patient survival in breast and colorectal cancer and variations among African American, Asian, and younger patients

**DOI:** 10.18632/oncotarget.28082

**Published:** 2021-09-28

**Authors:** Sofia Leaf, Lindsey Carlsen, Wafik S. El-Deiry

**Affiliations:** ^1^Laboratory of Translational Oncology and Experimental Cancer Therapeutics, The Warren Alpert Medical School, Brown University, Providence, RI 02903, USA; ^2^The Joint Program in Cancer Biology, Brown University and the Lifespan Health System, Providence, RI 02903, USA; ^3^Department of Pathology and Laboratory Medicine, The Warren Alpert Medical School, Brown University, Providence, RI 02903, USA; ^4^Cancer Center at Brown University, The Warren Alpert Medical School, Brown University, Providence, RI 02903, USA; ^5^Pathobiology Graduate Program, The Warren Alpert Medical School, Brown University, Providence, RI 02903, USA; ^6^Hematology-Oncology Division, Department of Medicine, Rhode Island Hospital and The Warren Alpert Medical School, Brown University, Providence, RI 02903, USA; ^*^These authors contributed equally to this work

**Keywords:** breast cancer, colorectal cancer, BRCA1, biomarker, early onset

## Abstract

Breast cancer (BC) and colorectal cancer (CRC) are common and show poor survival in advanced stages. Using The Cancer Genome Atlas (TCGA) computational tool cBioPortal, we evaluated overall patient survival in BRCA1 mRNA-low versus -high cohorts (<−1.29 versus >1.05 SD from mean BRCA1 expression, respectively). Analysis included 1082 BC patients with mRNA data (PanCancer Atlas), 382 CRCs (Firehose Legacy) and 592 CRCs (PanCancer Atlas). As previously reported, BRCA1 mRNA-low tumor expression positively correlated with BC patient survival but was negatively associated in CRC. We observed a correlation between BRCA1 mRNA-high and age <45 years at CRC diagnosis using a Fisher’s exact test [Firehose Legacy database (*p*-value = 0.0091); CRC PanCancer Atlas (*p*-value = 0.0778)]. We correlated BRCA1 mRNA-low expression and basal BC (*p*-value = 0.0016) and BRCA1 mRNA-low tumors and frequency of African American patients (*p*-value = 0.0448) with BC. Other trends included higher frequency of advanced lymph node stage and mucinous adenocarcinoma among BRCA1 mRNA-low CRC and higher frequency of males in BRCA1 mRNA-high BC and CRC. African Americans more frequently had BRCA1 mRNA-low BC and BRCA1 mRNA-high CRC and the opposite was observed among Asians. Using a gene co-expression tool (cBioPortal), we observed TOP2A and ATAD5 levels correlate (Spearman’s correlation>0.6) with BRCA1 in BC and CRC, whereas LMNB2 correlates with BRCA1 in CRC, suggesting tissue-specific BRCA1 interactions. Our results indicate potential for BRCA1 mRNA expression levels as a prognostic biomarker in BC and CRC, suggest tissue-specificity in BRCA1 molecular interactions, and point to BRCA1 mRNA-high levels as a characteristic of CRC tumors in younger versus older individuals.

## INTRODUCTION

Breast cancer (BC) is the most common cancer in women besides nonmelanoma skin cancer. 12% of women will be diagnosed in their lifetime and the 5-year survival rate is as low as 28% once BC becomes metastatic [1]. There are five molecular subtypes of BC which differ in their molecular presentation: luminal A (hormone receptor (HR)+, HER2–), luminal B (HR+, HER2–/+), triple-negative (HR–, HER2–), HER2-enriched (HR–, HER2+), and normal-like, which is similar to luminal A. Triple-negative breast cancer can be further classified into basal-like 1, basal-like 2, immunomodulatory, mesenchymal, mesenchymal-stem like, luminal androgen receptor, or unstable. Each subtype differs in overall prognosis [2].

Colorectal cancer (CRC) is the third leading cause of cancer deaths and like BC, is especially deadly when it metastasizes (5-year survival rate at this point is ~13%). Though vast improvements in screening and development of new therapies have reduced CRC-related mortality, the incidence of CRC has actually been rising among young people over the past several decades [3]. Very little is known about the cause of this increase, either on a population or molecular level. Some hypotheses have been presented such as cancer-promoting changes in the microbiome due to unhealthy diet and sedentary lifestyle or obesity, but little has been done as far as investigating the molecular basis of this or any other mechanism [4].

Both BC and CRC are treated with surgery, chemotherapy, radiation, and in some cases, immunotherapy [5, 6]. BC is also treated with hormone ablative therapy such as tamoxifen [6]. Though effective, these treatments can cause significant toxicity that can have detrimental and rarely fatal effects on the patient. There is much interest in the field of cancer research regarding development of biomarkers to individualize treatment of existing therapies [7, 8]. Establishing and validating prognostic biomarkers can allow physicians and patients to make informed decisions that limit exposure of patients to unnecessary treatment and/or ensures that high-risk patients get the treatment they need.

Some biomarkers exist for BC and CRC patients. Three of the most notable biomarkers in BC are HRs such as estrogen receptor (ER), progesterone receptor (PR), and/or HER2/Neu overexpression/amplification, and BRCA1/2 mutations. However, even though these biomarkers have been significant prognostic advancements, they do have limitations. There are many different subtypes within BC that have unique molecular profiles, which increases the need for specificity and sensitivity of biomarkers. ER and HER2/Neu in particular, though widely used, still require additional mechanisms to be sufficiently effective due to shortcomings in approaches to estimation [9]. Moreover, in colorectal cancer, while mutations in genes such as KRAS, NRAS and BRAF have been associated with a worse prognosis, there is discrepancy over whether or not any truly reliable biomarkers have yet been clinically implemented [10].

BRCA1 (BReast CAncer gene) is a well-known tumor suppressor gene that functions as part of a complex to repair double strand DNA breaks. When BRCA1 is mutated, DNA double strand breaks remain unrepaired by homologous recombination which contributes to genomic instability that may result in transformation of cells and further tumor evolution [11–13]. BRCA1 is mutated in less than 1–7% of BC tumors when patients are not selected for family history [14, 15], but when altered it is a robust biomarker for BC susceptibility with mutation carriers having a lifetime risk of up to 85% for BC [1, 16, 17]. BRCA1 mutations also predict worse overall survival compared to patients harboring wild-type BRCA1 tumors [15]. BRCA1 mutations in CRC often result in loss of heterozygosity (LOH), have been found in almost 50% of sporadic cases [16], and offer similar utility as a biomarker for worse outcomes [18, 19] although conflicting results have been reported [16].

Prior work has evaluated the prognostic significance of high or low BRCA1 mRNA expression in BC and CRC. BC patients with BRCA1 mRNA-low expression are more likely to respond to anthracycline-based therapy [20] and have improved overall survival despite a significant correlation between BRCA1 mRNA-low expression and high histological grade [21]. One previous study found that high HER2/low BRCA1-expressing tumors were less sensitive to radiotherapy, leading to worse prognosis [22]. However, the effect of HER2 in this study could complicate making any conclusions as far as BRCA1 expression and prognosis in BC. Furthermore, low BRCA1 protein expression may be a biomarker for worse prognosis in colorectal cancer [12, 23, 24]. Compared to genomic profiling to identify mutations in the genome and loss of heterozygosity, evaluation of protein expression in patient samples using immunohistochemistry may be more accessible in a clinical setting [24]. Based on these suspected opposing effects of low BRCA1 expression on outcomes in BC and CRC, we investigated the value of BRCA1 mRNA-low versus -high expression as a prognostic biomarker in these cancers and further investigated differences in age, sex, tumor stage, metastasis score, tumor size and subtype, and race across these patient cohorts.

## RESULTS

### Low expression of BRCA1 mRNA in breast cancer patients correlates with better survival

**Figure 1:**
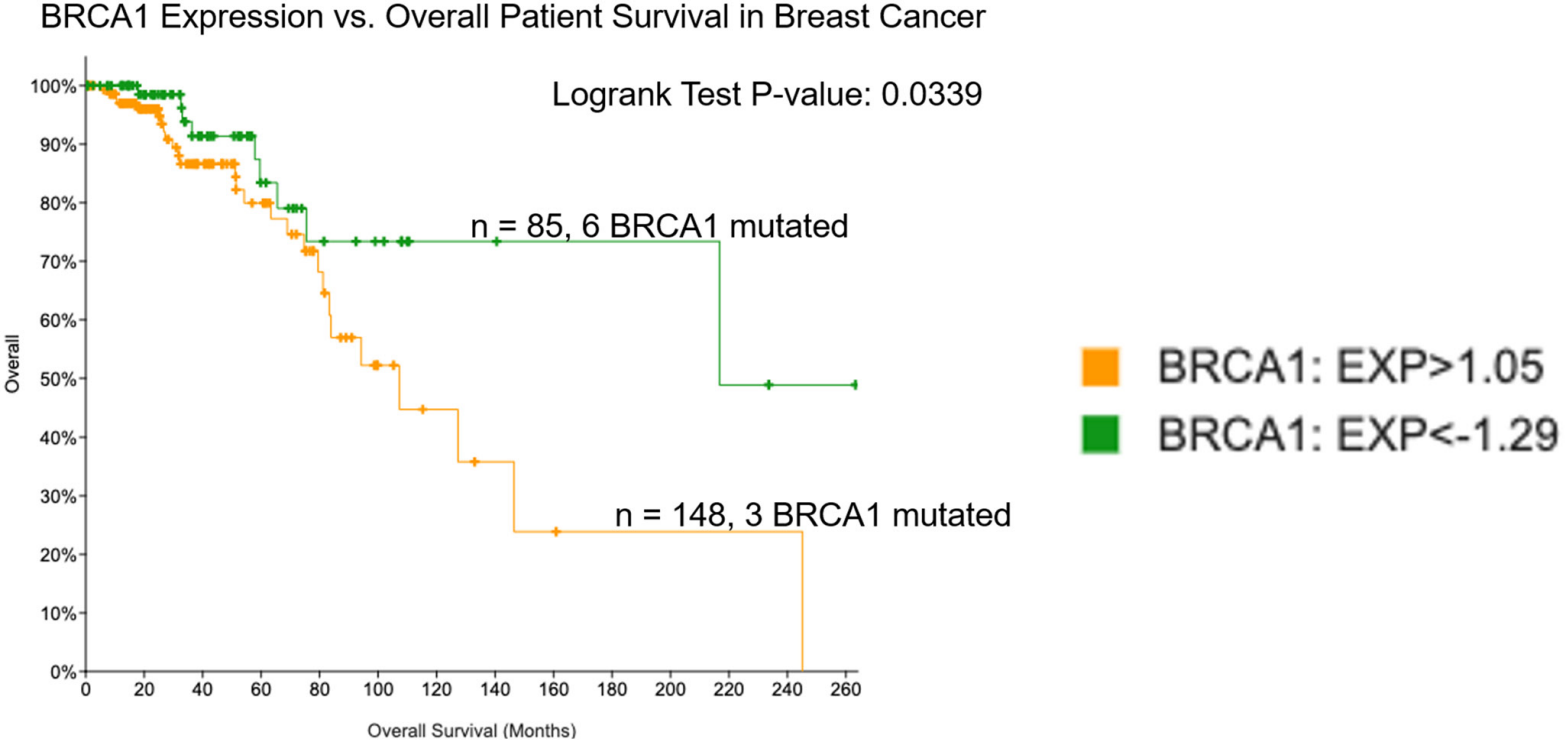
Low expression of BRCA1 in breast cancer correlates with improved overall survival. Patient survival was evaluated in groups expressing low versus high levels of BRCA1 mRNA using the TCGA Breast Invasive Carcinoma PanCancer database in cBioPortal.

It is known that BRCA1 mutations lead to worse outcomes in breast cancer and there is data to suggest that BC patients with low BRCA1 expression may have better overall survival and response to treatment [20, 21]. BRCA1 is a Tumor Suppressor Gene (TSG) that produces TSG proteins. These proteins play a role in preserving genetic material and helping DNA repair. The connection between BRCA1 mutations and worse BC survival rates is thought to be due to the fact that any damage to the gene will affect its ability to aid in repairing damaged DNA which can lead to further mutations and cancerous cells [12]. Thus, we hypothesized that low BRCA1 expression levels would impact on patient survival in BC. To investigate this, we used cBioPortal to establish groups of patients with low versus high expression of BRCA1 and evaluated the difference in overall survival between these two groups. We found that among 1082 patients in the Breast Invasive Carcinoma PanCancer Atlas database, 85 patients expressed low (<−1.29 standard deviation from the mean of all samples) levels of BRCA1 and 148 patients expressed high (>1.05 standard deviation from the mean of all samples) levels of BRCA1.These groups had significantly different (*p*-value < 0.05) overall survival, with low expression predicting better prognosis (Figure 1).

### Subtype and race distribution vary across BRCA1 mRNA-low versus BRCA1 mRNA-high breast cancer

**Figure 2:**
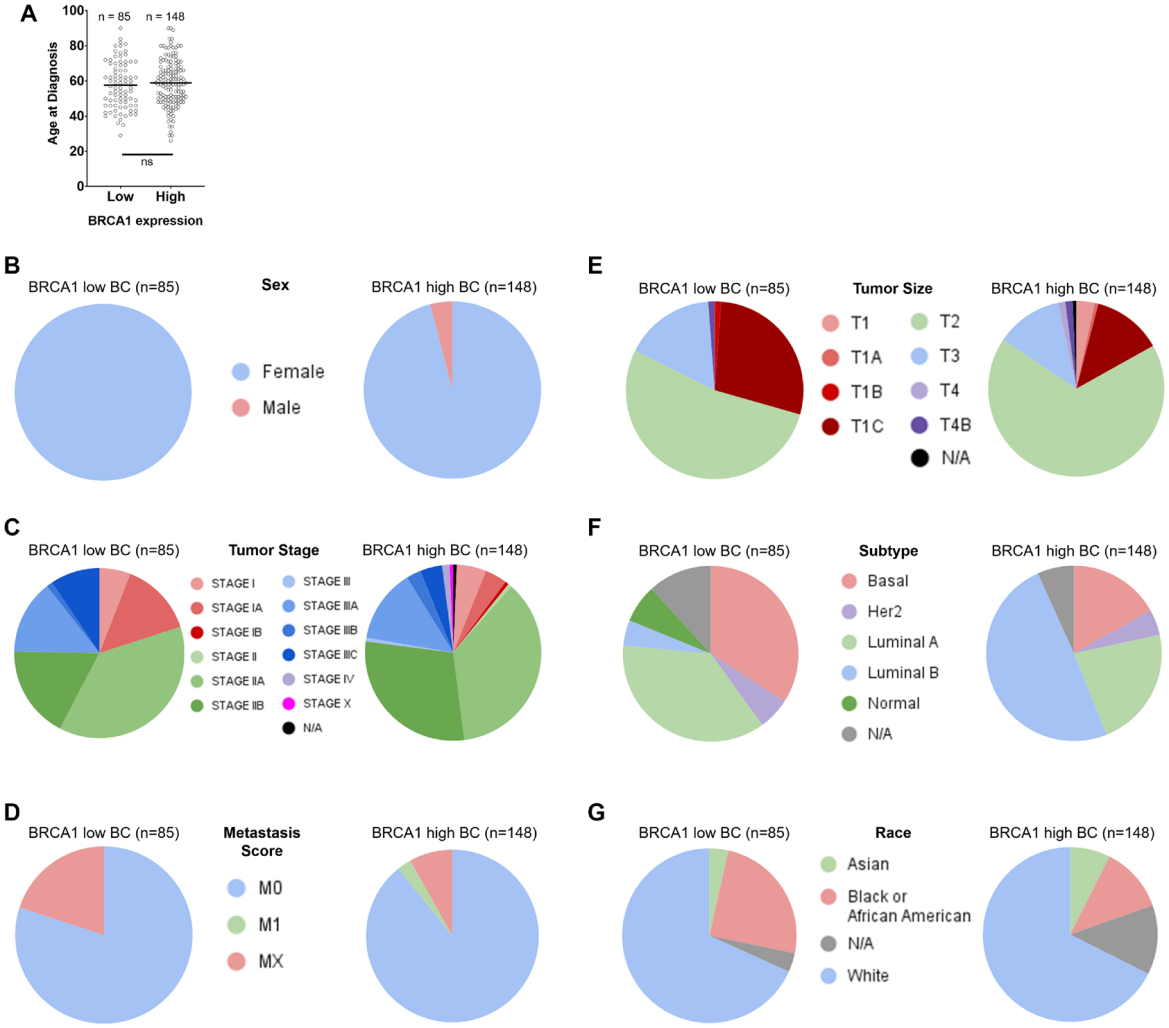
Distribution of age, sex, tumor stage, metastasis score, tumor size, subtype, and race across BRCA1 low versus high groups in breast cancer. Age (**A**), sex (**B**), tumor stage (**C**), metastasis score (**D**), and tumor size (**E**) were relatively evenly distributed across BRCA1 mRNA-low versus -high groups. Subtype (**F**) and race (**G**) distribution showed some notable differences across BRCA1 mRNA-low versus -high groups.

To investigate BRCA1 mRNA-low versus -high BC patient populations further, we evaluated distribution of age, sex, tumor stage, metastasis score, tumor size, subtype, and race. We found that age (Figure 2A), sex (Figure 2B), tumor stage (Figure 2C), metastasis score (Figure 2D), and tumor size (Figure 2E) were relatively evenly distributed across BRCA1 mRNA-low versus -high groups. This data is also displayed in Table 1. It is notable that 6 male breast cancer patients appeared in the BRCA1 mRNA-high group, whereas none appeared in the BRCA1 mRNA-low group, although this may be due to the larger sample size of BRCA1 mRNA-high patients. Subtype (Figure 2F) and race (Figure 2G) distribution showed some notable differences across BRCA1 mRNA-low versus -high groups.

**Table 1:** Distribution of sex, tumor stage, metastasis score, tumor size, race, subtype, and lymph node stage (for CRC only) across BRCA1 mRNA-low versus -high groups in BC and CRC

		BC		CRC	
		BRCA1 low (*n* = 85)	BRCA1 high (*n* = 148)	BRCA1 low (*n* = 38)	BRCA1 high (*n* = 51)
**Sex**	**Female**	85/85 (100%)	142/148 (96%)	18/38 (47%)	17/51 (33%)
	**Male**	0/85 (0%)	6/148 (4%)	20/38 (53%)	33/51 (65%)
	**N/A**	0/85 (0%)	0/148 (0%)	0/38 (0%)	1/51 (2%)
**Tumor Stage**	**I**	17/85 (20%)	15/148 (10%)	4/38 (11%)	5/51 (10%)
	**II**	47/85 (55%)	98/148 (66%)	10/38 (26%)	19/51 (37%)
	**III**	21/85 (25%)	31/148 (21%)	11/38 (29%)	15/51 (29%)
	**IV**	0/85 (0%)	2/148 (1%)	9/38 (24%)	8/51 (16%)
	**X**	0/85 (0%)	1/148 (1%)	0/38 (0%)	0/51 (0%)
	**N/A**	0/85 (0%)	1/148 (1%)	4/38 (11%)	4/51 (8%)
**Metastasis Score**	**M0**	68/85 (80%)	132/148 (89%)	19/38 (50%)	31/51 (61%)
	**M1**	0/85 (0%)	4/148 (3%)	8/38 (21%)	9/51 (18%)
	**MX**	17/85 (20%)	12/148 (8%)	9/38 (24%)	10/51 (20%)
	**N/A**	0/85 (0%)	0/148 (0%)	2/38 (5%)	1/51 (2%)
**Tumor Size**	**T1**	25/85 (29%)	25/148 (17%)	1/38 (3%)	1/51 (2%)
	**T2**	45/85 (53%)	100/148 (68%)	4/38 (11%)	4/51 (8%)
	**T3**	14/85 (17%)	18/148 (12%)	24/38 (63%)	40/51 (80%)
	**T4**	1/85 (1%)	4/148 (3%)	7/38 (18%)	5/51 (10%)
	**Tis**	0/85 (0%)	0/148 (0%)	1/38 (3%)	0/38 (0%)
	**N/A**	0/85 (0%)	1/148 (1%)	1/38 (3%)	1/51 (2%)
**Race**	**White**	58/85 (68%)	100/148 (68%)	29/38 (76%)	33/51 (65%)
	**Black or African American**	21/85 (25%)	18/148 (12%)	4/38 (11%)	13/51 (26%)
	**Asian**	3/85 (4%)	11/148 (7%)	3/38 (8%)	2/51 (4%)
	**N/A**	3/85 (4%)	19/148 (13%)	2/38 (5%)	3/51 (6%)
**BC Subtype**	**Luminal A**	31/85 (36%)	33/148 (22%)		
	**Luminal B**	4/85 (5%)	73/148 (49%)		
	**Basal**	29/85 (34%)	25/148 (17%)		
	**Her2**	5/85 (6%)	7/148 (5%)		
	**Normal**	6/85 (7%)	0/148 (0%)		
	**N/A**	10/85 (12%)	10/148 (7%)		
**Lymph Node Stage**	**N0**			16/38 (42%)	29/51 (57%)
	**N1**			11/38 (29%)	12/51 (24%)
	**N2**			10/38 (26%)	9/51 (18%)
	**N/A**			1/38 (3%)	1/51 (2%)
**CRC Subtype**	**Colon Adenocarcinoma**			20/38 (53%)	35/51 (69%)
	**Rectal Adenocarcinoma**			9/38 (24%)	11/51 (22%)
	**Colorectal Adenocarcinoma**			0/38 (0%)	1/51 (2%)
	**Mixed**			2/38 (5%)	1/51 (2%)
	**Mucinous**			7/38 (18%)	3/51 (6%)

Values correlate with those used to generate charts in Figures 2 and 4.

Evaluation of subtype across BRCA1 mRNA-low versus -high groups revealed that the basal subtype was more frequent in the BRCA1 mRNA-low group (Figure 2F). This relationship was statistically significant based on a Fisher’s exact test of the contingency table shown in Supplementary Table 1. The basal subtype of BC is generally more aggressive than the luminal and Her2 subtypes and makes up a significant portion of all triple negative BC (TNBC) cases. This was surprising, as we found that BRCA1 mRNA-low BC patients actually had better overall survival compared to BRCA1 mRNA-high BC patients (Figure 1). This could be explained by the fact that the BRCA1 mRNA-low versus -high groups had substantially different proportions of luminal A versus luminal B cases (Figure 2F). As the luminal A subtype has the best prognosis in BC, and the BRCA1 mRNA-low group had a larger proportion of luminal A patients, it is likely that this can explain the differences in overall survival [25], however the impact of subtype on differential patient outcomes across BRCA1 mRNA-low versus -high patients is unclear from this data.

Distribution of race also varied across BRCA1 mRNA-low versus -high patient populations in BC, with African American patients more frequently falling into the BRCA1 mRNA-low group (25% low versus 12.2% high) and Asian patients more frequently falling into the BRCA1 mRNA-high group (7.5% high versus 3.6% low) (Figure 2G). The relationship between BRCA1 mRNA levels and frequency of African American patients was statistically significant based on a Fisher’s exact test of the contingency table shown in Supplementary Table 1. As African American women are more likely to get BC (particularly TNBC) [26], this result is surprising because we found that low expression of BRCA1 mRNA correlated with better outcomes in BC (Figure 1).

### Low mRNA expression of BRCA1 in colorectal cancer patients correlates with poor survival

**Figure 3:**
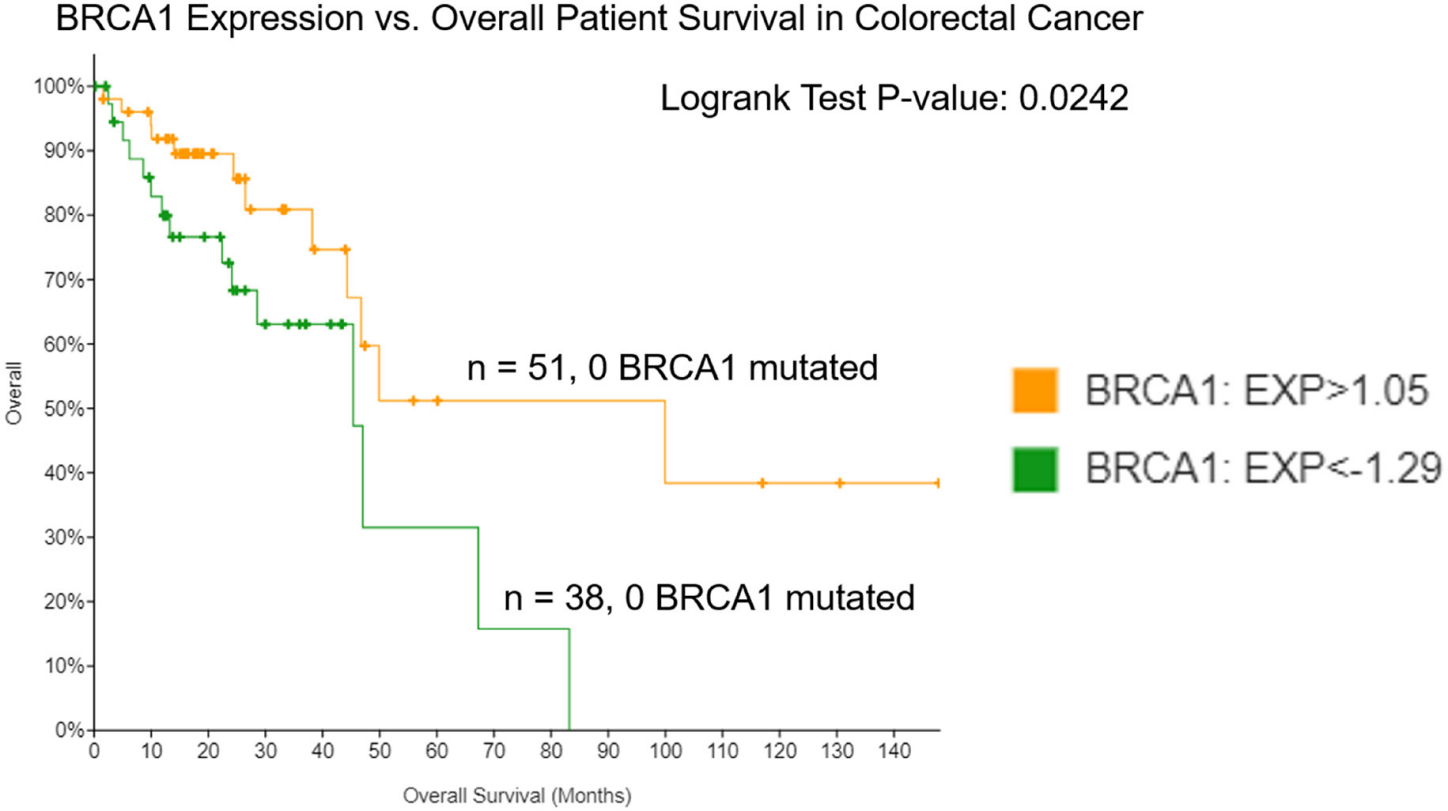
Low expression of BRCA1 in colorectal cancer correlates with worse overall survival. Patient survival was evaluated in groups expressing low versus high levels of BRCA1 mRNA using the TCGA Colorectal Adenocarcinoma Firehose Legacy database in cBioPortal.

BRCA1 mutations not only predict development of breast and ovarian cancer [27, 28] but may also predict early onset CRC cancer, and it has been previously described that low BRCA1 protein expression may be a biomarker for worse prognosis in colorectal cancer [12, 23, 24]. Thus, we sought to confirm these findings on the mRNA transcript level and further investigate differences between the BRCA1 mRNA-low versus -high groups that may explain differential patient survival between them. We evaluated the effect of BRCA1 expression on overall patient survival in CRC using TCGA and cBioPortal. We found that among 382 patients in the Firehose Legacy database, 51 expressed high (> 1.05 standard deviation from the mean of all samples) levels of BRCA1 mRNA and 38 expressed low (<−1.29 standard deviation from the mean of all samples) levels of BRCA1 mRNA. These groups had significantly different (*p*-value < 0.05) overall survival, with low expression of BRCA1 mRNA correlating with poor overall survival (Figure 3).

### Frequency of advanced lymph node stages and mucinous adenocarcinoma subtype is higher in BRCA1 mRNA-low versus -high colorectal cancer

**Figure 4:**
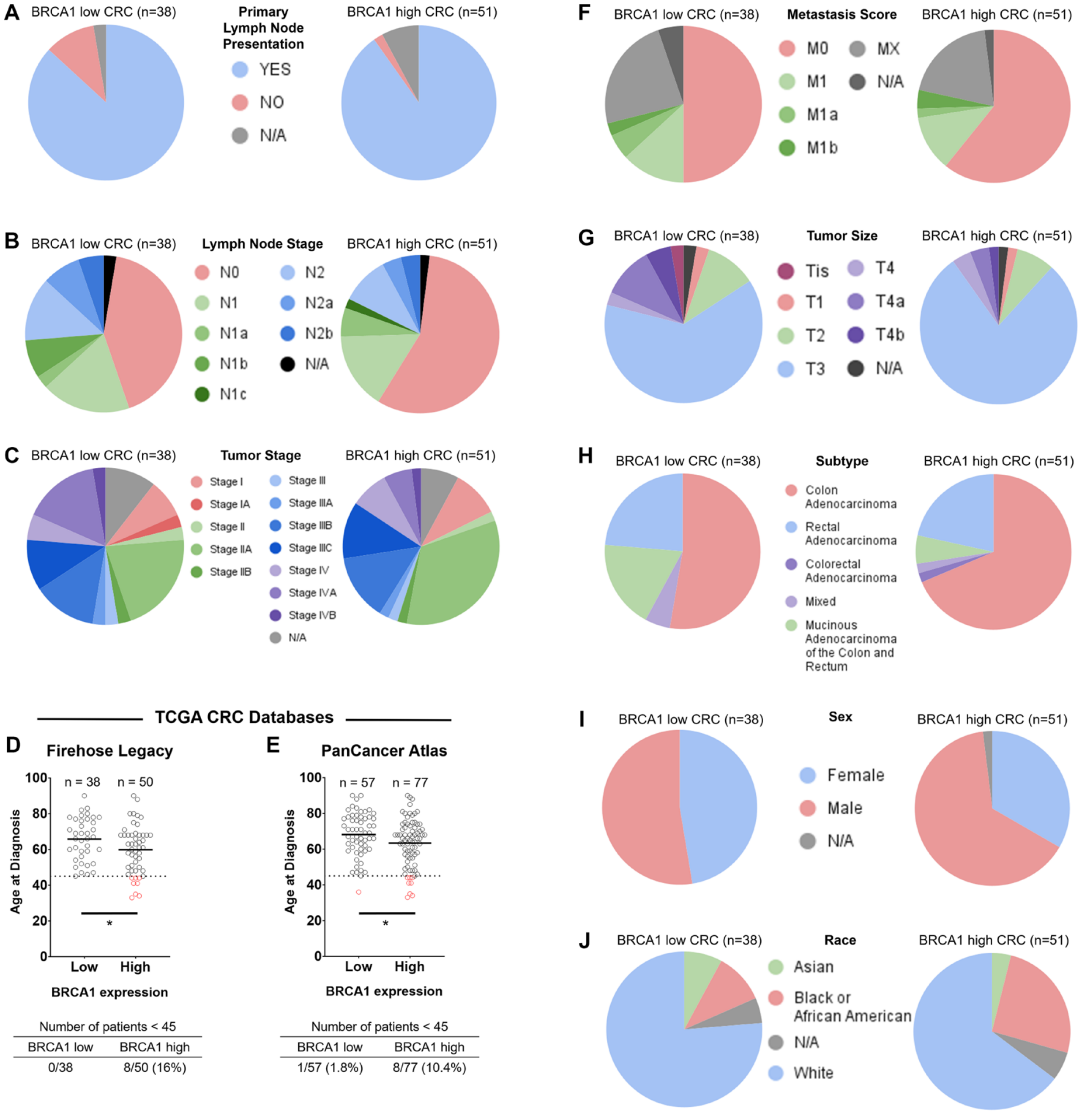
Distribution of primary lymph node presentation, lymph node stage, tumor stage, age, metastasis score, tumor size, specific cancer type, sex, and race across BRCA1 mRNA-low versus -high groups in colorectal cancer. Primary lymph node presentation (**A**) was relatively evenly distributed across BRCA1 mRNA-low versus -high groups. Lymph node stage (**B**), tumor stage (**C**), age (**D** and **E**), metastasis score (**F**), tumor size (**G**), specific cancer type (**H**), sex (**I**), and race (**J**) showed some noteworthy differences across BRCA1 mRNA-low versus -high groups. Patients <45 years old are indicated by red data points in (D–E). ^*^= *p*-value < 0.05.

To investigate these patient populations further, we evaluated distribution of age, sex, tumor stage, metastasis score, tumor size, lymph node stage, and race across BRCA1 mRNA-low versus -high CRC. Primary lymph node presentation was relatively evenly distributed across BRCA1 mRNA-low versus -high groups (Figure 4A). Lymph node stage (Figure 4B), tumor stage (Figure 4C), age (Figure 4D and 4E), metastasis score (Figure 4F), tumor size (Figure 4G), specific cancer type (Figure 4H), sex (Figure 4I), and race (Figure 4J) showed some noteworthy differences across BRCA1 mRNA-low versus -high groups. The frequency of these factors across BRCA1 mRNA-low versus -high groups can be found in Table 1.

Compared to the BRCA1 mRNA-high group, the BRCA1 mRNA-low group of CRC patients had a higher proportion of tumors in more advanced lymph node stages (N1/N2) (Figure 4B), a slightly higher frequency of patients with stage IV CRC (Figure 4C), a slightly higher metastasis score (Figure 4F), and had a greater frequency of mucinous adenocarcinomas (Figure 4H). These are unfavorable characteristics in terms of patient outcomes [29]. The BRCA1 mRNA-low group also had a higher percentage of large (T4) tumors, but the majority of patients in both the BRCA1 mRNA-low and -high groups still fell into large tumor size (T3/T4) categories, making it difficult to make conclusions regarding tumor size (Figure 4G). Together, these findings are in line with our finding that low BRCA1 mRNA in CRC is associated with worse patient outcomes. These findings also highlight the tendency of mucinous adenocarcinomas to have low BRCA1 mRNA expression, the mechanism for which remains to be elucidated. As BRCA1/2-mutated CRC tumors are more frequently of the mucinous adenocarcinoma subtype compared to BRCA1/2 wild-type tumors, this finding further suggests a role for the loss of BRCA1 activity in the development of mucinous adenocarcinoma [29].

### Frequency of male and African American patients is higher in BRCA1 mRNA-high versus low CRC

Whereas the distribution of lymph node stage, tumor stage, metastasis score, and CRC subtype across BRCA1 expression levels are in line with our findings that low BRCA1 mRNA correlates with worse outcomes, the distribution of sex and race across these groups is more difficult to explain. For example, the frequency of males is higher in the BRCA1 mRNA-high group compared to the BRCA1 mRNA-low group (Figure 4I). It is recognized that there is a survival advantage for females in CRC due to a protective effect of female hormones [30]. It may be that there is a mechanism by which high BRCA1 levels compensate indirectly or directly for the lack of protective female hormones in males, and future investigation should evaluate levels of relevant hormones in male colorectal cancer patients across BRCA1 mRNA-low versus -high groups. It is notable that the frequency of male patients is also higher in the BRCA1 mRNA-high group in breast cancer (Figure 2B), which may instead support a possible interaction between male hormones and BRCA1 that promotes the development of these cancers.

African American CRC patients tend to have lower overall survival in CRC compared to other races [31]. However, we found that African American patients more frequently fell into the BRCA1 mRNA-high group (Figure 4J), which had better outcomes in CRC (Figure 3). Worse outcomes in African American patient populations are largely attributed to socioeconomic reasons, but differential survival is still seen after adjustment for these factors [31]. The biological drivers of this disparity is a topic of current investigation, and our results may suggest a role of high BRCA1 expression in counteracting these mechanisms.

These findings reinforce the importance of evaluating multiple factors that contribute to patient survival in cancer, as in this case the effect of metastasis score, tumor size, lymph node stage, and specific cancer type likely contribute more significantly to patient survival compared to the sex and race effects.

The molecular mechanisms responsible for the correlation of low BRCA1 levels and poor patient survival in CRC remains elusive. One possible explanation is that low BRCA1 expression may be a marker of the methylator phenotype, in which areas of the genome, particularly surrounding tumor suppressors such as BRCA1, are silenced via methylation. In CRC, the methylator phenotype often co-occurs with BRAF mutations, which predict worse prognosis compared to tumors with wild-type BRAF [32]. Due to limited patient numbers, we were unable to evaluate the effect of BRAF mutations on overall survival in the context of BRCA1 expression.

### Younger patients (<45-years old) are more likely to have BRCA1 mRNA-high versus -low CRC

The incidence and mortality of CRC among young people has been rising in recent decades despite an overall decrease in incidence and mortality of CRC in the overall population. Most sources mention an increase in sedentary lifestyle, unhealthy western diets, obesity, microbiome and smoking among young people as potential contributing factors to this increase but very little progress has been made as far as uncovering molecular mechanisms that may explain this phenomenon [33]. Using two TCGA Colorectal Adenocarcinoma databases (Firehose Legacy and PanCancer Atlas) and the computational tool cBioPortal, we calculated the average age of CRC diagnosis in BRCA1 mRNA-low versus -high groups. We found that there was a statistically significant lower age of CRC diagnosis in the BRCA1 mRNA-high group in both databases (65.79 vs. 59.84 in Firehose Legacy; 68.18 vs. 63.39 in PanCancer Atlas) (Figure 4D and 4E). We further evaluated the age distribution of CRC cancer patients in the BRCA1 mRNA-low versus -high groups. Each TCGA database had a higher frequency of young (<45 years old) patients in the BRCA1 mRNA-high group compared to the BRCA1 mRNA-low group (Figure 4D–4E, Supplementary Figure 1). A Fisher’s exact test using the contingency table shown in Supplementary Table 1 determined a statistically significant (*p*-value 0.0091) correlation between BRCA1 mRNA levels and age of diagnosis in the Firehose Legacy database but not the CRC PanCancer Atlas database (*p*-value = 0.0778).

There is much debate with regard to the prognosis of young colorectal cancer patients compared to older patients. Younger patients tend to present with more advanced disease, but may be more resilient when faced with toxicities associated with harsh cancer treatments such as chemotherapy and radiation [34–36]. As we found that high expression of BRCA1 correlated with better outcomes in CRC, our findings may support the hypothesis that younger patients tend to benefit more after treatment. Our findings also suggest that despite a survival advantage of BRCA1 mRNA-high CRC, increased levels of this tumor suppressor might directly or indirectly contribute to the development of CRC in young people. Further investigation with larger databases containing larger sample sizes of young patients should investigate the role of BRCA1 expression specifically in this patient population.

### Differential relationship between BRCA1 mRNA levels and cancer type across African American and Asian patient populations

In order to investigate possible reasons for differential patient survival across BRCA1 mRNA low versus high groups in BC and CRC, we evaluated the relative proportion of different races across these groups as it is known that African American patients are more likely to get CRC and are more likely to die from this disease [37]. We found that in BC, African American patients tended to express low levels of BRCA1 mRNA whereas Asian patients tended to express high levels of BRCA1 mRNA (Figure 2G). The relationship between African American patients and BRCA1 mRNA-low levels in BC was significant (*p*-value = 0.0448) according to a Fisher’s exact test of the contingency table shown in Supplementary Table 1. In CRC, however, African American patients tended to express high levels of BRCA1 mRNA whereas Asian patients tended to express low levels of BRCA1 mRNA (Figure 4J). We expected that in BC, the relatively larger proportion of African American patients within the BRCA1 mRNA-low group would have a negative effect on the overall survival of this group compared to BRCA1 mRNA-high patients. However, we found the opposite relationship. Together, these results indicate an impact of race on tissue-specific expression of BRCA1 that correlates significantly with differential patient survival. There is little to no existing literature on this topic, suggesting that little to no previous work has been done to explain this relationship. Further investigation of these results is needed in order to potentially personalize treatment based on race and tumor BRCA1 expression levels.

### TOP2A and ATAD5 correlate with BRCA1 mRNA in BC and CRC, whereas LMNB2 correlates with BRCA1 only in CRC

**Figure 5:**
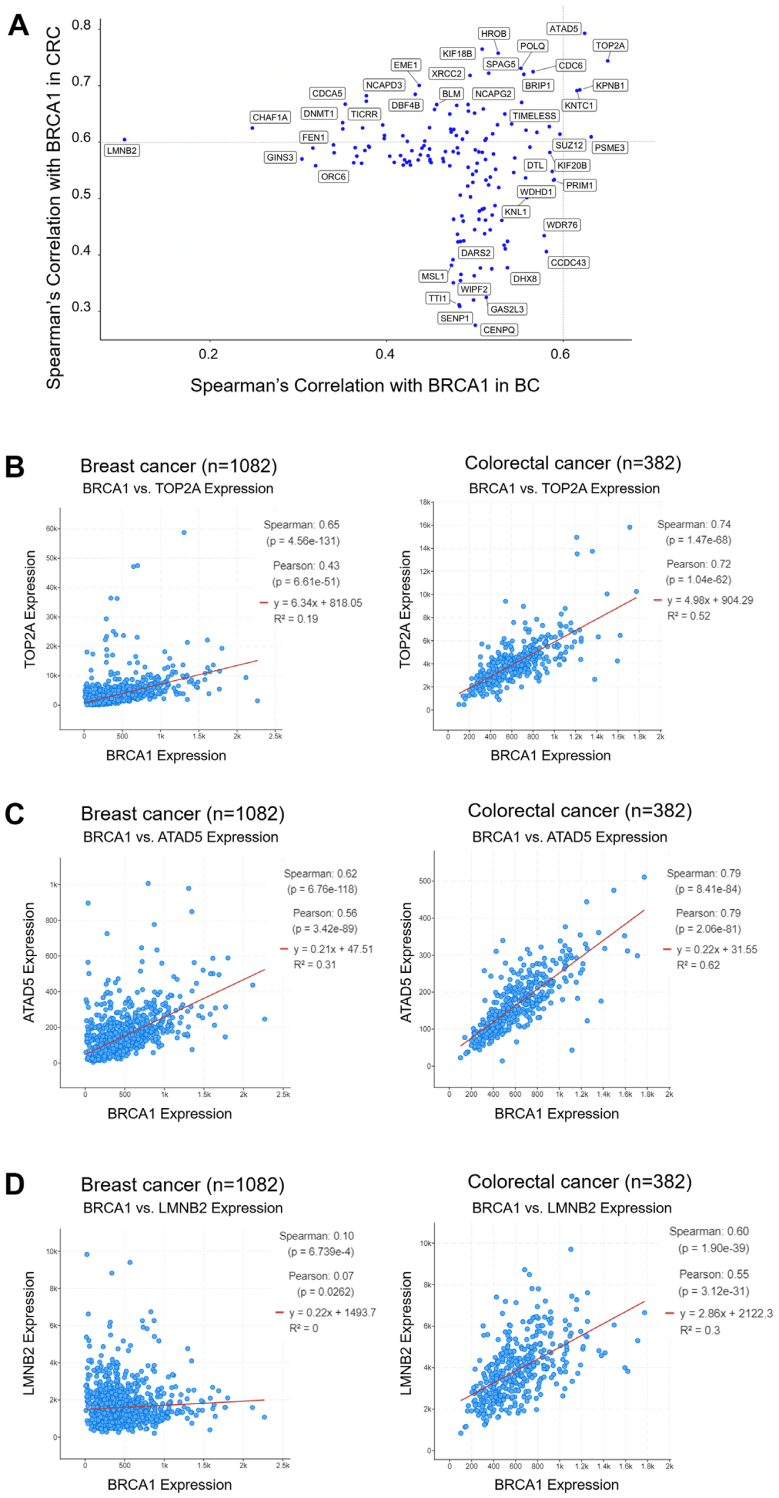
TOP2A and ATAD5 mRNA expression correlates with BRCA1 mRNA expression in BC and CRC, whereas LMNB2 expression only correlates with BRCA1 expression in CRC. The top 100 genes whose expression correlated to BRCA1 expression were selected for BC and CRC and plotted on a scatter plot of Spearman’s correlation with BRCA1 in BC versus CRC (**A**). TOP2A (**B**) and ATAD5 (**C**) mRNA expression correlates with BRCA1 mRNA expression across BC and CRC. LMNB2 mRNA expression correlates with BRCA1 mRNA expression only in CRC (**D**).

In addition to evaluating differential distribution of age, sex, tumor stage, metastasis score, tumor size/subtype, and race across BRCA1 mRNA-low versus -high groups, we also identified genes whose expression correlated with BRCA1 mRNA expression across tissue type or in a tissue-specific manner. We used the BC PanCancer Atlas and CRC Firehose Legacy databases, and using the cBioPortal co-expression tool identified the top 100 genes which correlated with BRCA1 mRNA expression in each cancer type. Both positive and negative correlations were included in the selection of the top 100 genes for each cancer type, but we found that there were no negative correlations. These two 100-gene lists were combined and overlapping transcripts were removed to generate a list of 156 genes which contained the top 100 for both cancer types. These genes were plotted on a scatter plot according to their Spearman’s correlation with BRCA1 mRNA in BC (x-axis) and CRC (y-axis) (Figure 5A). A Spearman’s correlation of 0.6 as a cutoff for significance, is denoted by the dotted line in the Figure.

We identified some genes that correlated with BRCA1 expression in both BC and CRC, such as TOP2A (DNA Topoisomerase IIα) (Figure 5B) and ATAD5 (ATPase Family AAA Domain Containing 5) (Figure 5C). It may be that in BC, low expression of BRCA1 correlates with improved outcomes due to the co-occurring low expression of TOP2A, an enzyme needed for DNA replication. As loss-of-function mutations in mammalian ATAD5 cause genomic instability and tumorigenesis, it is possible that low expression of BRCA1 in CRC correlates with worse outcomes due to co-occurring low expression of ATAD5. The role of TOP2A in CRC and the role of ATAD5 in BC are more difficult to explain.

Surprisingly, there were no genes that correlated with BRCA1 mRNA expression uniquely in BC, but there were several genes that correlated with BRCA1 expression uniquely in CRC. One of these genes was LMNB2 (Lamin B2). Lamins serve as a layer on the nuclear membrane and aid in providing structure for the nuclear envelope which contains proteins that play a role in gene regulation. LMNB2 is a chromatin remodeling protein that also plays a role in eukaryotic cell proliferation by organizing the nuclear membrane during mitosis. Chen-Hua Dong et al. recently found that high expression of LMNB2 in CRC tumors correlates with worse disease-free cumulative and overall survival, and that LMNB2 promotes the progression of colorectal cancer by silencing p21 expression [38]. In line with this, we found that young CRC patients, who tend to express high levels of BRCA1 in their tumors, also tend to express low levels of p21 and this may be due to simultaneous high expression of LMNB2 (Supplementary Table 2). Despite this, we found that high BRCA1 mRNA levels correlate with better outcomes in CRC (Figure 3). It is possible other BRCA-mediated mechanisms counteract the negative impact of p21 suppression by LMNB2.

## DISCUSSION

Our novel observations include correlation between BRCA1 mRNA-high tumor expression and age <45 years at CRC diagnosis, BRCA1 mRNA-low expression and basal BC, mucinous adenocarcinoma among BRCA1 mRNA-low CRC, and higher frequency of males in BRCA1 mRNA-high BC and CRC. African Americans more frequently had BRCA1 mRNA-low BC and BRCA1 mRNA high CRC and the opposite was observed among Asians. TOP2A and ATAD5 levels correlated with BRCA1 expression in BC and CRC, whereas LMNB2 correlated with BRCA1 in CRC, suggesting tissue-specific BRCA1 interactions.

BRCA1 mutations correlate with worse prognosis in BC and CRC and several studies suggest a possible impact of BRCA1 expression on patient survival. Thus, we evaluated the impact of low versus high BRCA1 mRNA expression on patient survival across these cancer types using publicly available TCGA. We found that BRCA1 mRNA-low expression correlated with better survival in BC but worse prognosis in CRC. These findings are expected based on previous work [12, 20–24]. Our direct comparison across tissue type contributes to the idea that there is tissue specificity in the impact of loss of BRCA1 expression on patient outcomes.

Our results indicate BRCA1 mRNA-low CRC tended to be more advanced in tumor, metastasis, and lymph node stages, possibly explaining the worse overall survival seen in this group. Results in BC were more difficult to explain. While age, sex, tumor and metastasis score, and tumor size were relatively evenly distributed across BRCA1 mRNA-low versus -high groups, subtypes were unevenly distributed in a way that made it difficult to make any conclusions. Investigation of race in these groups showed that African American patients tended have BRCA1 mRNA-low BC, which is surprising as we found the BRCA1 mRNA-low levels correlated with better outcomes and African American patients are known to have lower overall survival compared to other racial groups. It is very likely that factors not evaluated here due to lack of data available (including treatments received) might further explain these findings. It is also possible that the correlation of BRCA1 mRNA-low levels with increased patient survival in BC may be explained by the fact that BRCA1 is predominantly expressed in S-phase of the cell cycle. Therefore, it may be that high levels of BRCA1 are more likely to be expressed in proliferating BC cells. Additionally, BRCA1 can interact with p53 to induce DNA repair proteins such as DDB2 and inhibit p53-induced cell death [39]. As 60% of the BRCA1 mRNA-high BC patients harbored wild-type p53 in the tumors (data not shown), this mechanism could contribute to the worse overall survival observed in BC patients expressing high levels of BRCA1 mRNA.

Particularly interesting was the high frequency of African American patients in the BRCA1 mRNA-low group in BC compared to the high frequency of this group in the BRCA1 mRNA-high category in CRC. This suggests an impact of race on tissue-specific expression of BRCA1 that to our knowledge has not been previously described.

Evaluation of age distribution across CRC patients expressing low versus high levels of BRCA1 mRNA revealed a higher frequency of young (<45-years old) patients in the BRCA1 mRNA-high group. To our knowledge, this is the first report of BRCA1 as a potentially contributing factor to the increasing incidence and mortality of CRC in young people. With little existing literature proposing the molecular mechanisms behind this increase, it is difficult to assign a probable role of BRCA1 expression in promoting CRC specifically among young people. Further validation and investigation is needed to determine if there are mechanisms which contribute to the rise in CRC that are separate from the known risk factors such as sedentary lifestyle, obesity, smoking, microbiome or other factors.

It has been reported that BRCA1 is upregulated by DNA damage, then reduced to below basal levels in a p53-dependent manner [40]. In line with this finding, a large proportion (8/9) of CRC patients under the age of 45, a majority of whom expressed high levels of BRCA1 mRNA, harbored *TP53* mutations in their tumors (Supplementary Table 2). Our lab has also previously reported that BRCA1 contributes to cell-cycle arrest by transactivation of p21 in CRC cell lines through both p53-independent and p53-dependent mechanisms [41–43]. Interestingly, here we observe that young/BRCA1 mRNA-high CRC patients also more frequently expressed low levels of p21 compared to older/BRCA1 mRNA-low CRC patients (Supplementary Table 2). This may be due to the correlation of BRCA1 mRNA expression with LMNB2 expression specifically in CRC (Figure 5D), as LMNB2 has been shown to silence p21 expression [38].

We previously demonstrated that loss of BRCA1 can contribute to the aggressiveness of HRAS-driven BC *in vitro* and *in vivo* [44]. This raises the question as to whether a similar mechanism might exist in CRC, in which KRAS/NRAS are mutated up to 50% of the time [45], to result in worse survival of CRC patients with low BRCA1 expression levels. One might envision CRC with low BRCA1 and KRAS/NRAS mutations to be more invasive and angiogenic as we previously observed in the BC models.

While the status of BRCA1 and BRCA2 both play a major role in determining BC susceptibility and work together to protect the genome, these proteins have distinct functions. BRCA1 has many functions including checkpoint activation and DNA repair, while BRCA2 functions mainly in homologous recombination [46]. We found that high BRCA2 mRNA expression was more frequent in young CRC patients (who also tend to express BRCA1 mRNA-high tumors) compared to older CRC patients. A similar relationship between BRCA1 and BRCA2 was observed in BC (Supplementary Table 2). This is not surprising, as BRCA1 and BRCA2 are coordinately regulated in mammary cells [47], and may indicate that both BRCA1 and BRCA2 play a role in the age, sex, race, subtype, and stage effects in CRC and BC.

Limitations of this analysis include limited patient sample sizes available in TCGA after filtering for low or high mRNA expression of BRCA1, which is likely why we observed drastic changes in the *p*-value of Kaplan-Meier curves when the mRNA expression cutoffs were adjusted at small intervals. A limitation of TCGA is the lack of treatment data, and so it is possible that some effects seen here are due to differences in treatment received. Another limitation of the analysis is overlap among cases between the Firehose Legacy and PanCancer databases as is evident from the age distribution of the youngest CRC patients with BRCA1 mRNA-high expression in the tumors (Supplementary Figure 1A and 1B).

Together, our results indicate the potential for BRCA1 expression as a prognostic biomarker in BC and CRC, suggest tissue-specificity in the impact of loss of BRCA1 expression as related to patient outcomes, and may reveal high levels BRCA1 as a molecular characteristic among younger patients with CRC. We believe we are the first to evaluate differences in patient demographics and clinical characteristics directly across these two BC and CRC groups to reveal their potential relationship with BRCA1 expression. Among our most important findings is a possible relationship between high BRCA1 expression and young age of colorectal cancer diagnosis, providing a starting point to investigate the puzzling rise in colorectal cancer incidence and mortality among young people for which little explanation exists. We also identify a relationship between BRCA1 expression and race which could inspire investigation to explain a similarly elusive disparity of African American patients compared to other races in the incidence and mortality of breast cancer and colorectal cancer.

## MATERIALS AND METHODS

### Kaplan-Meier curves

We used cBioPortal to create Kaplan Meier curves (Figures 1 and 3) to evaluate loss of expression of BRCA1 in tumors and patient survival outcomes in BC and CRC. We used the Breast Invasive Carcinoma database (The Cancer Genome Atlas (TCGA), PanCancer Atlas) which contained 1082 total patients with mRNA data, and the Colorectal Adenocarcinoma database (TCGA, Firehose Legacy) which contained 382 samples with mRNA data. Additional age distribution analysis was conducted using the Colorectal Adenocarcinoma database (PanCancer Atlas) which contained 592 samples with mRNA data. For all analyses, mRNA expression z-scores were relative to all samples. High and low cutoffs used for generation of Kaplan-Meier curves were >1.05 and <−1.29 standard deviation from the mean of all samples, respectively. Each curve created by cBioPortal has its corresponding Logrank Test *P*-Value, and if that value was less than 0.05, the separation between the lines was considered significant. Both Kaplan Meier curves and raw data were obtained on 8/26/2021.

### Patient population analysis

Information about the patients included in the BRCA1 high and BRCA1 low groups such as age, sex, tumor stage, metastasis score, tumor size and subtype, and race were evaluated using the OncoPrint tab in cBioPortal. Age distribution graphs were generated using GraphPad. Identification of genes whose expression correlated to BRCA1 in BC and/or CRC, including their Spearman correlation value and *p*-values, was completed using the co-expression tab in cBioPortal and the resulting lists were used to generate a scatter plot in R.

### Statistical analysis

An unpaired *t*-test was used to calculate statistical significance of difference in the mean age across BRCA1 low versus high groups in Figure 2A and Figure 4D and 4E. A *p*-value of < 0.05 was considered significant and is denoted by an asterisk (^*^). To determine the statistical significance of the association between BRCA1 levels and age, sex, tumor stage, metastasis score, tumor size, tumor subtype, or race, a Fisher’s exact test was used and a *p*-value of < 0.05 was considered significant.

## SUPPLEMENTARY MATERIALS


