# Supplementary material for: Opposing effects of BRCA1 mRNA expression on patient survival in breast and colorectal cancer and variations among African American, Asian, and younger patients
Source: Oncotarget. 2021 Sep 28;12(20):1992–2005. doi: 10.18632/oncotarget.28082 (PMC8487727; doi:10.18632/oncotarget.28082)
Supplement: Supplementary file 1 [file oncotarget-12-1992-s001.pdf]

Opposing effects of BRCA1 mRNA expression on patient survival in breast and colorectal cancer and variations among African American, Asian, and younger patients

SUPPLEMENTARY MATERIALS

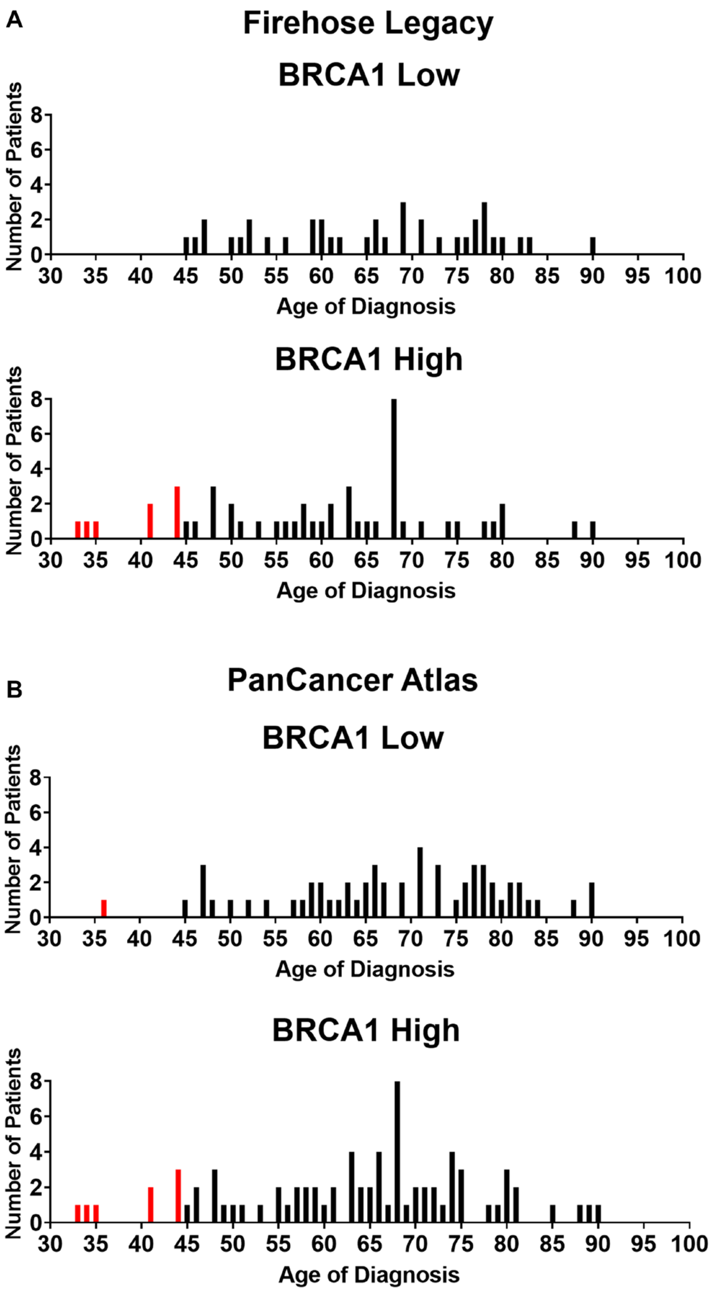

Supplementary Figure 1: Frequency of young (<45 year old) patients is higher in BRCA1 high vs. BRCA1 low colorectal cancer. Patients in two TCGA databases (Firehose Legacy and PanCancer Atlas) were separated based on BRCA1 mRNA expression (low = <−1.29 standard deviation from the mean of all samples; high = >1.05 standard deviation from the mean of all samples). The ages of patients in these groups are shown with patients <45 years old indicated in red.

Supplementary Table 1: Contingency tables used to calculate statistical significance of the correlation between BRCA1 levels and frequency of African American patients/BC subtype (top) or age of CRC diagnosis (bottom) using a Fisher's Exact Test

BC PanCancer Atlas		
BC Subtype	Patients with low BRCA1 mRNA	Patients with high BRCA1 mRNA
Basal	29	25
Non-basal	46	113
	p-value = 0.0016	
Race	Patients with low BRCA1 mRNA	Patients with high BRCA1 mRNA
Black or African American	21	18
Non-black or African American	61	111
	p-value = 0.0448	
**CRC Firehose Legacy**		
Age of Diagnosis	Number of patients with low BRCA1 mRNA	Number of patients with high BRCA1 mRNA
<45	0	8
>45	38	42
	p-value = 0.0091	
**CRC PanCancer Atlas**		
Age of Diagnosis	Number of patients with low BRCA1 mRNA	Number of patients with high BRCA1 mRNA
<45	1	8
>45	56	69
	p-value = 0.0778	

Some patients do not have data on subtype or race and were excluded in this analysis.

Supplementary Table 2: Frequency of BRCA1, BRCA2, p21, Rb low/high expression and TP53, ATM mutations in BC and CRC patient cohorts

CRC Firehose Legacy								
	BRCA1		BRCA2		p21		Rb	
Age	mRNA Low	mRNA High	mRNA Low	mRNA High	mRNA Low	mRNA High	mRNA Low	mRNA High
<45 (*n* = 8)	0/8 (0%)	8/8 (100%)	0/8 (0%)	6/8 (75%)	3/8 (38%)	1/8 (13%)	2/8 (25%)	1/8 (13%)
>45 (*n* = 80)	38/80 (48%)	42/80 (53%)	18/80 (23%)	20/80 (25%)	13/80 (16%)	13/80 (16%)	12/80 (15%)	11/80 (14%)

CRC PanCancer Atlas										
	BRCA1		BRCA2		p21		Rb		TP53	ATM
Age	mRNA Low	mRNA High	mRNA Low	mRNA High	mRNA Low	mRNA High	mRNA Low	mRNA High	Mutated	Mutated
<45 (*n* = 9)	1/9 (11%)	8/9 (89%)	1/9 (11%)	5/9 (56%)	3/9 (33%)	0/9 (0%)	3/9 (33%)	0/9 (0%)	8/9 (89%)	1/9 (11%)
≥45 (*n* = 125)	56/125 (45%)	69/125 (55%)	29/125 (23%)	25/125 (20%)	19/125 (15%)	21/125 (17%)	22/125 (18%)	22/125 (18%)	70/110 (64%)	14/110 (13%)

BC PanCancer Atlas			
BRCA1		BRCA2	
mRNA Low	mRNA High	mRNA Low	mRNA High
85/233 (36%)	148/233 (64%)	41/233 (18%)	65/233 (28%)

Frequency of TP53 and ATM mutations are included for the PanCancer Atlas database (all 9 patients <45 years old were profiled for mutations, and 110/125 patients >45 years old were profiled for mutations). BRCA1 low/high expression is defined as <−1.29/>1.05 standard deviation from the mean, respectively. BRCA2, p21, and Rb low/high expression is defined as <−1/>1 standard deviation from the mean, respectively.
